# Psychosocial interventions to support the mental health of informal caregivers of persons living with dementia – a systematic literature review

**DOI:** 10.1186/s12877-021-02020-4

**Published:** 2021-02-01

**Authors:** Henrik Wiegelmann, Sarah Speller, Lisa-Marie Verhaert, Liane Schirra-Weirich, Karin Wolf-Ostermann

**Affiliations:** 1 Institute for Public Health and Nursing Research, Health Sciences Bremen, University of Bremen, Grazer Straße 4, 28359 Bremen, Germany; 2 Department of Social Services, Centre for Participation Research, Catholic University of Applied Sciences of North Rhine-Westphalia, Robert-Schuman-Straße 25, 52066 Aachen, Germany

**Keywords:** Dementia, Informal caregiver, Mental health, Psychosocial interventions, Systematic review

## Abstract

**Background:**

Informal caregivers of persons living with dementia have an increased risk of adverse mental health effects. It is therefore important to systematically summarize published literature in order to find out which mental health interventions generate effective support for informal caregivers of persons living with dementia. The objective of this study is to conduct a systematic review of intervention content, effectiveness and subgroup differentiation of mental health interventions for informal caregivers of persons with dementia living at home.

**Method:**

We searched four electronic databases (PubMed, PsychINFO, Scopus and CINAHL) and included only methodically high-quality randomized controlled trials (RCTs), published in English or German language between 2009 and 2018. The intervention programmes focused on mental health of family caregivers. A narrative synthesis of the included studies is given.

**Results:**

Forty-eight publications relating to 46 intervention programmes met the inclusion criteria. Burden, depression and quality of life (QoL) are the predominant parameters that were investigated. Twenty-five of forty-six interventions (54.3%) show positive effects on at least one of the outcomes examined. Most often, positive effects are reported for the outcome subjective burden (46.2%). Only six studies explicitly target on a certain subgroup of informal dementia caregivers (13%), whereas all other interventions (87%) target the group as a whole without differentiation.

**Conclusion:**

The most beneficial results were found for cognitive behavioural approaches, especially concerning the reduction of depressive symptoms. Besides this, leisure and physical activity interventions show some good results in reducing subjective caregiver burden. In order to improve effectiveness, research and practice may focus on developing more targeted interventions for special dementia informal caregiver subgroups.

**Supplementary Information:**

The online version contains supplementary material available at 10.1186/s12877-021-02020-4.

## Background

Dementia has been recognized as a global public health issue, posing challenges on different societal levels ranging from the individual and families to communities and governments [1–3]. Worldwide, there are approximately 50 million persons living with dementia. Every year about 10 million incident diagnoses are made and current projections assume that by 2050 about 132 million persons globally will live with dementia [1–3]. Informal caregivers - predominantly family but also friends - provide a majority of dementia care, estimated to represent 40% of the total cost of dementia worldwide [4]. As models suggest, this unpaid work is mainly performed by women, contributing to 71% of the global hours of informal dementia care work [5]. Most of dementia care is provided in the private sphere, with differences according to the world region. For instance, this applies to 69% of dementia care in high-income countries (World Bank classification) like the USA, Japan, Australia or Germany, whereas these numbers are much higher in countries with lower gross national income (93–98%). In a global perspective, the estimated proportion of persons with dementia cared for at home is 84% [5].

The motives for becoming a caregiver of a person with dementia are diverse, influenced by, among other things, traditional gender roles, dyadic relationship constellations, the housing situation, socio-economic resources or cultural influences. A considerable proportion of dementia caregivers (men 33%; women 39%) indicate that they had no choice but to become an informal caregiver. Further reasons are, for instance, the wish to protect and enhance the wellbeing of the person in need, a sense of obligation to repay the care received as children, care as an extension of the existing caring role within a romantic relationship [6]. However, it should also be stressed/pointed out that informal caregiving can also be perceived in a positive way, i.e. causing a sense of personal accomplishment and gratification, feelings of mutuality in a dyadic relationship, an increase of family cohesion and functionality or a sense of personal growth and purpose in life [7].

By taking over more comprehensive care and support tasks successively over time, due to the progressive course of dementia, the likelihood of health-related problems increases for informal caregivers [8]. Many informal caregivers face challenges in multiple domains, covering physical, social, financial and mental health strains [9–12]. Several studies show that mental health aspects play a central role in the overall health of informal dementia caregivers [10, 13, 14]. For instance, the risk for depression and anxiety increases as a result of everyday stress [15]. Likewise, informal caregivers express an increased perceived burden or a reduction in QoL [16, 17]. Moreover, a deterioration of the informal caregiver’s mental health increases the likelihood of institutionalization [9], followed by negative effects on QoL [18] and social participation of the person with dementia [19]. Even if the institutionalization can help to reduce the daily care work for informal caregivers, the respective decision may also lead to feelings of guilt, anger, anxiety, depression as well as financial problems [20].

Compared with caregivers of persons with others diseases, mental health related indicators like distress and stress, burden or subjective wellbeing are particularly worse for dementia caregivers [21]. Studies suggest that this high burden on mental health is linked to the fact that dementia caregivers provide more care work in hours per week and assist with more numbers of activities of daily living (ADL) or instrumental activities of daily living (IADL) [17, 20]. Furthermore, behavioural changes specific for dementia may lead to higher levels of mental stress and caregiver depression [22]. If we look at the group of family caregivers, there is evidence of in-group heterogeneity in terms of vulnerability for mental health problems. Studies indicate that a number of factors increase vulnerability including: a) socio-demographic variables (female gender, spousal relationship, cohabitation, lower income/financial inadequacy), b) disease-related variables (frontotemporal dementia, duration of caregiving, more neuropsychiatric symptoms, behavioural problems, impairment in basic activities), c) caregiver variables (high level of neuroticism, high expressed emotion, less secure attachment style, low sense of confidence in caregiving role, high role captivity, emotion-based and confrontative coping strategies) as well as d) relationship factors like poorer relationship quality and low levels of intimacy [20].

As there is still no medical cure for dementia, psychosocial interventions to support dementia caregivers and persons living with dementia have gained more and more attention in recent years, with promising results, including in regard to strengthening mental health of caregivers [23–26].

This review aims to provide an update on high quality psychosocial intervention studies on mental health promotion for informal caregivers of persons living with dementia, describing intervention effects on key mental health outcomes. Additionally, the subgroup orientation of interventions is analysed, because it is still unclear whether mental health support measures are adequately tailored to the needs of specific socio-demographic subgroups of dementia caregivers [25]. This is of particular interest because the diversity of informal caregivers of persons living with dementia and their respectively diverse support needs are widely recognized. Distinctive and frequently mentioned factors in the literature are: age [14], gender relation [27], family/kinship relationship [28], housing situation [29] or professional activity of informal caregivers [30], ethnic background [31] or social environment [32]. Approaches focusing on particular subgroups of informal caregivers therefore might be more effective than more general interventions [25, 33, 34]. To the best of our knowledge, there is only one previous study examining psychosocial interventions and their effects in certain subgroups of informal caregivers of persons living with dementia [34]. Their main finding is that interventions work better it they focus on certain caregiver subgroups (i.e. female caregivers). However, they also point out that targeting subgroups has received little or no attention in research so far and that research must focus with more emphasis on the issue of tailoring psychosocial interventions to the needs of specific subgroups of dementia caregivers [34].

The aim of this analysis is to systematically review empirical evidence from high quality RCTs about non-pharmacological psychosocial interventions and their effectiveness focusing on major mental health parameters to promote the health of informal caregivers of persons with dementia living at home in the community. Furthermore, an analysis of the subgroup orientation of interventions is presented. Against this background, the following main questions are raised:

### Content and effects

- What kinds of interventions have been implemented to improve mental health outcomes of informal caregivers of persons living with dementia?
- What effects on mental health outcomes are described?

### Subgroups and effects

- What are the specific subgroups of informal caregivers the intervention programmes relate to?
- What effects are reported for interventions targeting a particular subgroup?

## Methods

The key methodical steps are documented using the PRISMA 2009 Checklist is provided [35]. The methodical approach includes the three steps search strategy, selection of articles and assessment of methodical quality.

### Search strategy

Papers were systematically searched and retrieved from the electronic databases PubMed, PsychINFO, Scopus and CINAHL. The searches and retrieval were carried out in August 2018. The following combined search terms were used on title and abstract: [(dementia OR Alzheimer*) AND (caregiver* OR care-giver* OR carer* OR home care OR home-based care OR community-dwelling OR domestic care OR relatives OR couple* OR spouse*)]. The combination of search terms was developed in English and then translated into German. Database searches were carried out in German and English, results were merged. Search strategies were developed by the project team and tailored to the databases. The full search enquiry, using the example of the PsychInfo database, is provided as a supplementary document accompanying this paper (Additional file 1).

### Selection of articles

The literature selection was based on the list of inclusion and exclusion criteria summarized in Table 1. Studies were included if they investigate at least one of the clinical outcomes often applied in intervention research to support mental health of informal caregivers of persons living with dementia [23, 36–38]. Outcomes (depression, burden, quality of life, well-being, anxiety, stress, grief and mood) were specified before data extraction. We included only RCTs because they are widely considered to be the most rigorous method for assessing the efficacy of an intervention [39]. The interventions must aim primarily on supporting the mental health of informal caregivers of persons living with dementia. The reporting of the intervention programme must be sufficiently detailed with regard to content, duration, sessions/contacts, medium(s) used, location, group or individual approach, target group. Furthermore, only studies with high methodical quality according to criteria based upon Cochrane Collaboration Guidelines were included [9].

**Table 1:** Inclusion and exclusion criteria for literature search

Criteria	Inclusion	Exclusion
Population	• Informal caregiver and persons with diagnosis of dementia (dyads) (> 18 years) • Care takes place at home (home-based care) by informal caregivers	• Persons < 18 years • People in need of care with inborn disabilities • Professional caregiving
Intervention	• Programmes and services to promote mental health of informal caregiver of persons living with dementia • A detailed description of intervention (content, duration, sessions/contacts, follow-up, medium used, location, group/individual approach, target group)	• Pharmacological and bio-medical interventions • Further training of professional nursing and health professionals
Outcomes	• Validated measures of mental health • Evaluation of intervention effects with validated quantitative scales	
Study design & publication type	• Randomized controlled trials (RCT) • High methodological quality (min. 8 points) [9] • Published in peer-review journals	• Grey literature
Years	• 2009–2018	• Publications prior to 2009
Language	• German and English-language studies	• Other languages

Two reviewers (H.W., S.S.) independently screened a random sample of 166 titles and abstracts in which they were blinded to authors and journal titles, and reached strong agreement on the application of the eligibility criteria (Cohen’s κ = 0.83). Again, two reviewers independently screened all titles and abstracts and reviewed full-text articles considered for inclusion (H.W., S.S.). Detailed discussions in the research team (H.W., S.S., K.W.-O.) prepared and accompanied the full text analysis. To reach consensus a third opinion (K.W.-O.) was consulted in case of existing differences between the two main coders. An Excel workbook designed specifically for the organization and documentation of screening processes was used [40]. The screening process followed the Preferred Reporting Items for Systematic Reviews and Meta-Analyses (PRISMA) guidelines [35]. [Here: Figure summarizes the selection process.

### Assessment of methodical quality

Additionally, two reviewers (H.W., S.S.) independently rated the methodical quality of the studies included. Papers were evaluated using criteria established by Brodaty and colleagues based on Cochrane Collaboration Guidelines [9, 41]. This tool assesses five methodical domains (design, subjects, outcomes, statistics and results) for an appraisal of included studies (Table 2). The instrument yields a score between 0 and 11, indicating poor quality with less than five points. Papers were classified as being of good quality with a score of at least eight points.

**Table 2:** Criteria for rating the methodical quality of studies

Criterion	Score
Design	
Randomized	1
Controlled	1
Subjects	
Use of standardized diagnostic criteria	1
All subjects accounted for/withdrawals noted	1
Outcomes	
Well-validated, reliable measures	2
Questionable/unreliable outcome measures	0
Statistics	
Statistical significance considered	1
Adjustment for multiple comparisons	1
Evidence of sufficient power	1
Results	
Blind ratings	1
Follow-up 6 months or beyond	1
Good quality	> 7
Poor quality	< 5

Finally, we created three tables describing the results. The first (Additional file 2) shows the interventions included in the analysis regarding key characteristics like content, dosage, medium, features, recipients, group size, follow up, quality score and mental health outcomes. The content aspect plays an important role in the further course of the analysis, because individual studies are assigned to these umbrella categories and effects of the interventions on caregivers mental health is compared based on these categories. The interventions were classified into five main categories using the fundamental concepts or features applied and reported by the authors. In case of interventions with multiple components weighted similarly, the research team used qualitative coding procedure and made a consensual decision. Second, Table 3 focuses on the statistical significance of intervention results on relevant mental health outcomes given by the authors. Significant effects are defined as significantly stronger (*p* < 0.05) improvement in the intervention group than in the control group. Third, Additional file 3 gives an overview on the interventions that target a specific subgroup of informal caregivers of persons living with dementia.

**Table 3:** Effectiveness of intervention programmes on mental health of ICs of persons living with dementia

Study	Outcome categories							
	Depression	Burden	QoL	Well-being	Anxiety	Stress	Grief	Mood
**Psychoeducation (*n*** **= 20)**								
Berwig et al. [42]		+	–					
Blom et al.^a^ [43]	+	n. r.			+			
Chen et al. [44]		+						
Czaja et al. [45]	–							
Gitlin et al. [46]			–	–				
Gitlin et al. [47]	–	+		+				
Judge et al. [48]	+		–		+			
Kunik et al. [49]	–	–						
Kuo et al.^b^ [50, 51]	+		+					
Kurz et al. [52]	–		–					
Livingston et al.^b^ [53, 54]			+		+			
Martin-Carrasco et al. [55]		+	–	+				
Martin-Carrasco et al. [56]		–	–	–				
Prick et al. [57]	–	–						
Rotrou et al. [58]	+	–						
Soylemez et al. [59]	–	–	–					
Steffen et al. [60]	–							–
Tang et al. [61]		–				–		
Tremont et al. [62]	+	–	–					
Wang et al. [30]		+	+					
**Leisure and physical activity (*n*** **= 9)**								
Charlesworth et al. (RYCT)^c^ [63]			–		–	–		
Connell et al. [64]	–					–		
Danucalov et al. [65]			+					
Gitlin et al. [66]	–	–						
Hirano et al. [67]		+						
Lowery et al. [68]		+		–		–		
Mahdavi et al. [69]		+						
Moore et al. [70]	–							–
Woods et al. [71]			–	–	–	–		
**Counselling (*n*** **= 8)**								
Brijoux et al. [72]		–	+					
Fortinsky et al. [73]	–	–						
Gaugler et al. [74]						–		
Gavrilova et al. [75]		+	–			–		
Geschke et al. [76]	+		–					
Guerra et al.^d^ [77]		+	–			–		
Joling et al. [78]	–	–	–		–			
Phung et al. [79]	–		–					
**Cognitive behavioural approaches (*n*** **= 7)**								
Au et al. [80]	+							
Cheng et al.^a^ [81, 82]	+	+		–		+		
Kamkhagi et al. [83]	+	–	–					
Kwok et al. [84]		+						
Losada et al. (CBT) [85]	+				–			
Losada et al. (ACT) [85]	–				–			
Meichsner et al.^a^ [86, 87]	–	–		+			+	
**Befriending & Peer-support (*n*** **= 2)**								
Charlesworth et al. (CSP)^e^ [63]			–		–	–		
Laakkonen et al. [88]			–					

+: Results statistically significant. –: Results statistically not significant

^a^: Burden listed as outcome, but no results on burden reported (n.r.)

^b^: More than one publication but same intervention

^c^: Two different interventions are tested in Charlesworth et al. [63]. One of the interventions is “Remembering Yesterday Caring Today” (RYCT). This part of the study is classified here as “Leisure and physical activity”

^d^: Two different instruments used for the outcome stress, both without statistically significant results

^e^: The “Carer Supporter Programme (CSP)” is the second intervention tested in Charlesworth et al. [63]. This peer-to-peer approach is classified here as “Befriending and Peer-support”

As we know from a series of studies, the group of informal caregivers of persons living with dementia is characterized by heterogeneity in terms of various socio-demographic indicators such as age, sex, socio-economic status, ethnicity, kinship/family relation, housing situation or professional activity. These disparities between informal caregivers contribute to differences in health promotion needs and to the necessity to adapt intervention programmes to particular subgroups of caregivers [28, 34, 89]. With this in mind, we examined the interventions with regard to their subgroup orientation and checked systematically if the authors introduce a specific subgroup of informal caregivers and if they give a clear rationale for this focus, i.e. with regard to unique challenges the subgroup faces. Furthermore, we analysed whether, and if so, which parts (content, structure or procedure) of the intervention are adapted to the specific challenges of the target group and how. Finally, we reviewed the authors’ statements regarding the subgroup effectiveness of the interventions. We consider an intervention to be subgroup-oriented only if the authors explicitly elaborate on this. That means that the intervention programme must be rationally directed towards a specific informal caregiver subgroup and must aim to give explicit answers to particular challenges of that group identified by one or more socio-demographic characteristics (i.e. age, sex, kinship relation, housing situation, income, professional activity and ethnicity). This approach is similar to that of van Mierlo and colleagues who state that it needs more research into socio-demographic characteristics such as socioeconomic status, education or ethnic background [34]. In the present study, we take up this perspective and include the aspects mentioned in our narrative analyses. Owing to heterogeneity in study and intervention designs, we refrained from conducting a meta-analysis.

## Results

### Literature search

**Fig. 1:**
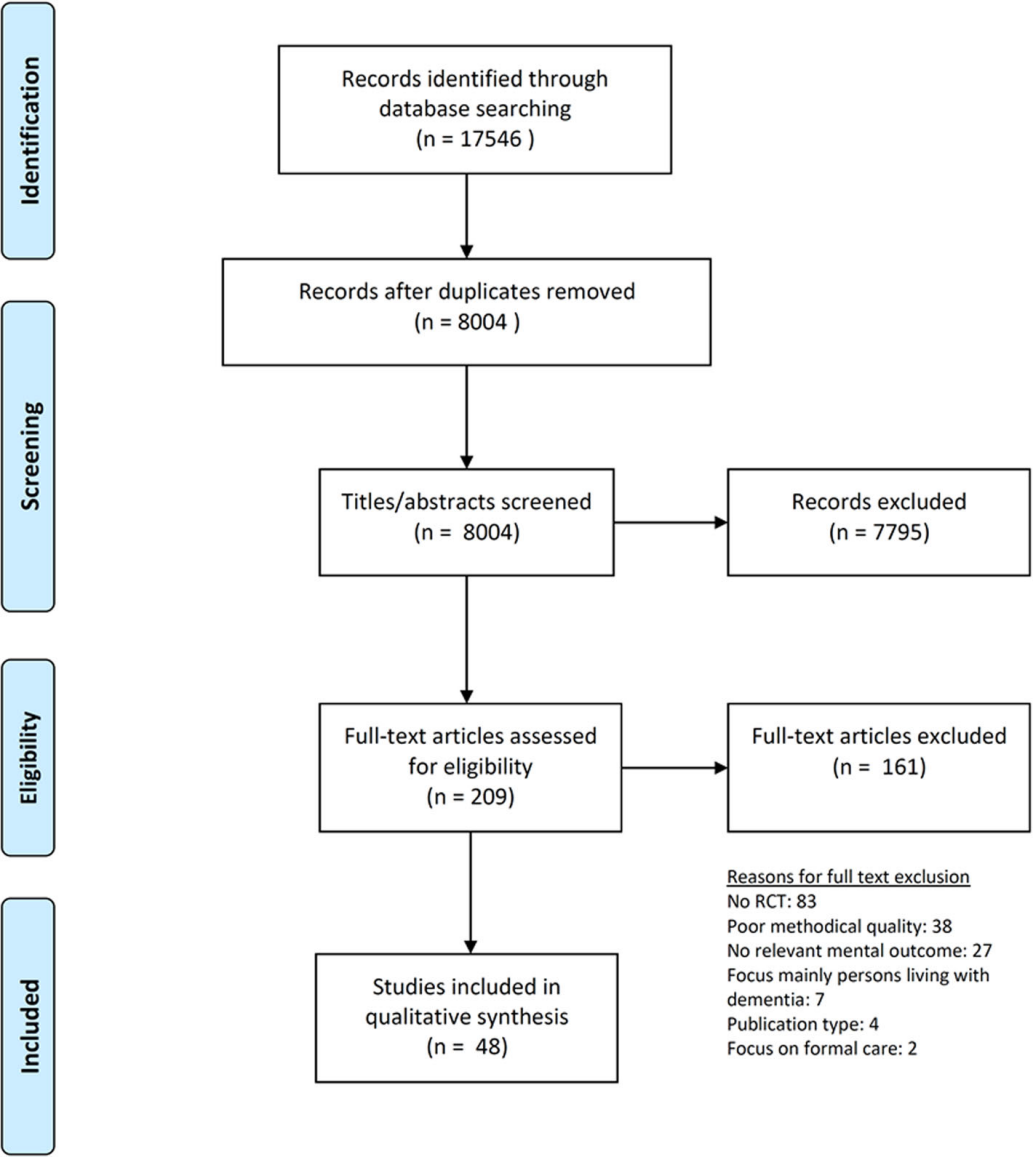
PRISMA flow diagram

Based on a broad search for the period 2009–2018 we identified 17,546 items, of which 9542 duplicates were automatically detected and excluded. In the course of a restrictive analysis of titles and abstracts, additional 7795 articles were rated as irrelevant and excluded from further analyses. In a next step, a total of 209 articles were subjected to a full text analysis and 161 items did not meet the inclusion criteria. Finally, 48 publications reporting on 46 unique intervention programmes met the selection criteria and were included in the classification and evaluation (synthesis). Four interventions are addressed in two separate articles each. Two other articles report on two separate intervention programmes. Overall, in 48 publications 46 separate interventions are described and tested. Figure 1 illustrates the selection and screening process using the PRISMA flow diagram [35].

### Intervention characteristics

Since the included interventions are rather diverse with regard to content and procedures implemented, a detailed presentation of intervention characteristics is provided in Additional file 2. Interventions differ mainly in terms of the fundamental concepts applied (content category), recipient groups, intensity (dosage) and the medium of delivery.

#### Content

Based on the fundamental concepts applied, the 46 unique intervention programmes were classified into five categories (total number and proportions in parentheses):

- Psychoeducation (20; 43.5%)
- Leisure and physical activity (9; 19.6%)
- Counselling (8; 17.4%)
- Cognitive behavioural approaches (7; 15.2%)
- Befriending and Peer-support (2; 4.3%)

#### Recipients

Informal caregivers of persons living with dementia are predominantly addressed individually (30; 65.2%) [30, 42–45, 47, 50, 51, 53–56, 58–65, 69, 70, 72, 73, 75, 77, 80–87]. Almost a quarter of the interventions follow a dyadic approach (12; 26%) [46, 48, 49, 52, 57, 66–68, 71, 76, 79, 88]. One intervention focuses on the family (1; 2.2%) [78] and three interventions (3; 6.5%) [63, 74] offer the recipients to choose between individual, dyadic or family approach.

#### Intensity

The interventions also vary in terms of intensity (dosage), which refers to the number of contacts or sessions between professional (i.e. therapist, social worker) or informal supporters (i.e. peer-support) and the informal caregivers over a certain period of time. Intervention intensity was rated according to the classification by Brodaty et al. [9] as follows: minimal (1–2 sessions), moderate (3–5 sessions), medium-high (6–10 sessions), or high/intensive (> 10 sessions). Twenty-one interventions (45.6%) can therefore be classified as high/intensive [42, 45–47, 50–52, 58, 60, 62–65, 67, 68, 71–73, 79, 83, 86, 87]. Further 19 interventions (41.3%) as medium-high [30, 43, 44, 48, 49, 53–57, 61, 66, 70, 74, 78, 80, 84, 85, 88]. There are five interventions (10.9%) with moderate intensity [59, 69, 75, 77, 81, 82], one (2.2%) with minimal intensity [76]. On average (median), an intervention in this sample has about eight (SD: 15.7) sessions/contacts over a period of about 16 (SD: 15.8) weeks.

#### Medium

By far the largest part of interventions are offered solely face-to-face (28; 60.9%) [30, 44, 48, 49, 52–59, 63, 65–69, 71, 73–78, 81–83, 85]. Nine other interventions (19.6%) combine face-to-face approaches with telephone sessions [42, 46, 47, 50, 51, 61, 63, 70, 72, 79]. Five interventions (10.9%) are designed exclusively as telephone support [62, 64, 80, 84, 86, 87]. There is also one intervention (2.2%) combining face-to-face, phone and web-based support [72], another one (2.2%) focusing on video conferences plus face-to-face [45], one (2.2%) working with a DVD/Video approach paired with phone support [60] and one intervention (2.2%) more that relies exclusively on internet support [43].

### Study characteristics

The studies vary with regard to the size of intervention and control group, time to follow-up, the overall methodical quality score and the mental health outcomes measured. By far the most studies come from the USA, followed by the United Kingdom, Germany and China.

#### Group size

The 44 separate studies involve a total of 6517 recipients (range 31–488, median = 128). Six studies (13.6%) involve less than 50 participants [44, 61, 65, 67, 83, 84], ten studies (22.7%) are in the range between 50 and 100 participants [30, 42, 59, 60, 72, 75–77, 80]. Further twelve interventions (27.3%) involve between 100 and 150 caregivers [45, 48, 50, 51, 55, 57, 68–70, 74, 81, 82, 85, 88], while four others (9.1%) involved between 150 and 200 persons [58, 64, 66, 78]. Six studies are in the range over 200 up to 250 (13.6%) [43, 46, 47, 49, 56, 62] and further six in the range over 250 randomized participants (13.6%) [52–54, 63, 71, 79, 86, 87].

#### Follow-up

Follow-up timing is classified as post-test, 3–5-month follow-up and six-months or more [3]. If measures were taken at multiple follow-up points, the last follow-up was assessed. About half of all studies (21, 47.7%) use a pre-post-test design [30, 43–45, 48, 50–52, 62, 63, 65, 67–69, 72–74, 78, 80–84]. For nine studies (20.5%), the latest post-test measurement time is between 3- and 6-months after the end of the intervention [42, 46, 47, 56–59, 61, 66]. Slightly less than every third study (14; 31.8%) has a follow-up point of six months or more [49, 53–55, 60, 64, 70, 71, 75–77, 79, 85–88].

#### Quality score

Only RCT studies of high methodical quality (see Additional file 2) with a score of at least eight points on the scale developed by Brodaty and colleagues were included in this review [9]. Of these 44 studies, 14 are rated with eight points (31.8%) [43–45, 48, 62, 64, 67, 69, 72, 73, 76, 80, 83, 84]. Twenty with nine points (45.5%) [30, 42, 47, 50–52, 55–61, 63, 65, 68, 71, 74, 81, 82, 85–87] and nine with ten points (20.5%) [46, 49, 53, 54, 66, 70, 75, 77, 78, 88]. One study reaches the maximum score of 11 points (2.3%) [79]. The average quality score is 8.9 points. Most studies score well in the domains design, subjects, and outcomes. Quite often, there are methodical weaknesses in the statistic domain, because adjustment for multiple comparisons and/or calculations for statistical power are not reported. The most frequent weaknesses are noted in the results domain. This is due to the fact, that a large number of intervention studies do not have a follow-up of at least 6 months after the end of the intervention phase, a minimum interval for studying the lasting effects of preventive interventions, according to the Society for Prevention Research [90].

#### Outcomes

A total of 102 relevant mental health parameter were tested across the 44 studies included and clustered into eight outcome groups. Burden (26) [30, 42–44, 47, 49, 55–59, 61, 62, 66–69, 72, 73, 75, 77, 78, 81–84, 86, 87] and depression (24) [43, 45, 47–52, 57–60, 62, 64, 66, 70, 73, 76, 78–83, 85–87] are the predominant outcomes studied. Followed by QoL (23) [30, 42, 46, 48, 50–56, 59, 62, 63, 65, 71, 72, 75–79, 83, 88]. Burden, depression and QoL are thus by far the most frequently investigated mental health outcomes. Stress is tested ten times [61, 63, 64, 68, 71, 74, 75, 77, 81, 82], well-being eight times [46, 47, 55, 56, 68, 71, 81, 82, 86, 87]. Anxiety is tested eight times as well [43, 48, 53, 54, 63, 71, 78, 85]. A small number of studies investigate the concepts of mood (2) [60, 70] and grief (1) [86, 87] as separate outcomes.

### Effects on informal caregivers by outcome

Due to their central position in discourse, we describe the outcomes burden, depression and QoL in separate chapters, the other outcomes are summarized in one section. In summary, the results on the effectiveness of the interventions are shown in Table 3. Overall, 34 out of the 102 measured outcomes show significant effects for the intervention groups, which means that positive effects were measured in a third of the mental health outcomes (33.3%).

### Subjective burden

In 26 interventions (56.5%), the impact on *subjective caregiver burden* is analysed. In 12 studies (46.2%), significant improvements for informal caregivers are observed. The most frequently used measurement tool is the Zarit Burden Interview, applied in 18 studies (69.2%).

#### Psychoeducation

Out of 20 psychoeducational interventions, 13 focus on measures of subjective caregiver burden. Five of these studies report significant effects for the intervention group [30, 42, 44, 47, 55]. Seven studies show no significant improvements for the intervention programme tested [49, 56–59, 61, 62]. Although mentioned as targeted outcome, one study does not report on the effects of the intervention on subjective caregiver burden [43].

#### Leisure and physical activity

Four studies working with leisure or physical activities investigate intervention effects on subjective burden of informal caregivers. Significant results are shown in three studies [67–69], while in one study no significant improvement is reported for the intervention group compared to the control group [66].

#### Counselling

Out of the five counselling interventions that focus on burden as an outcome, two report significant effects for the programme groups on subjective caregiver burden [75, 77]. The other three trials could not detect any significant improvements [72, 73, 78].

#### Cognitive Behavioural approaches

From the domain of cognitive-behavioural interventions, four studies examine the outcome caregiver burden. The results are inconsistent, while two studies report significant improvements for the intervention groups [81, 82, 84], two other studies do not find significant results in favour of the intervention [83, 86, 87].

#### Befriending and peer-support

There is no study in our sample from the field of befriending and peer-support examining the outcome subjective caregiver burden.

Overall, 12 out of 26 studies (46.2%) focusing on subjective caregiver burden, found statistically significant effects at the most recent follow-up point in comparison of intervention and control group. In relative terms, intervention programmes from the field of leisure and physical activity are the most successful. Three out of four studies (75%) show significant improvements, however, all three have a pre-post-test design and can therefore only confirm their effects in the short term.

It is important for the health of informal caregivers that interventions not only lead to short-term relief, but also contribute in the long term to an improvement in the mental health of informal caregivers. To verify this, caregiver interventions should include at least a follow-up measurement point of 6 months after the end of the intervention in order to investigate long-term effects [9]. Among the studies with significant results, three include a follow-up point of 6 months or more and thus can demonstrate long-term positive improvements [55, 75, 77].

#### Depression

The outcome *depression* was studied in 24 interventions (52.2%). Nine studies (37.5%) found statistically significant effects for depression. The predominant measurement tool used is the Center for Epidemiologic Studies Depression Scale (CES-D), implemented in 15 (62.5%) of these studies.

##### Psychoeducation

Eleven psychoeducational interventions provide information about the impact on caregiver depression. Four studies describe positive results, indicating improvements in depressive symptoms for caregivers in the intervention groups compared to participants in the control groups [43, 48, 50, 51, 58, 62]. Furthermore, seven studies report that the intervention provided, lead to no significant improvements in depressive symptoms [45, 47, 49, 52, 57, 59, 60].

##### Leisure and physical activity

Three studies assess whether leisure or physical activity support interventions have positive effects on depressive symptoms [64, 66, 70]. None of these interventions shows significant improvements for informal caregivers in the intervention groups.

##### Counselling

Counselling interventions do only slightly better. Among the four interventions available in this review [73, 76, 78, 79], only one intervention [76] showed significant improvements in terms of depression.

##### Cognitive Behavioural approaches

Four of six interventions, offering a variant of cognitive behavioural support, showed a positive effect [80–83, 85]. Two other interventions showed no significant effect [85–87].

##### Befriending and peer-support

There is no intervention in our sample from the field of befriending and peer-support examining the outcome depression.

#### Quality of life

Altogether, the authors of 23 studies (50%) report that they used validated instruments measuring *QoL* of informal caregivers. In five of these studies (21.7%), statistically significant effects are observed at the latest follow-up point. Most often, the Short Form-12 (SF-12) and Short Form-36 (SF-36) Health Questionnaires have been used.

##### Psychoeducation

Eleven studies from the field of psychoeducation investigate effects on the outcome *QoL*. Three studies report on interventions with positive effects [30, 50, 51, 53, 54], whereas eight studies measure no significant improvements for informal caregivers *QoL* [42, 46, 48, 55, 56, 59, 62].

##### Leisure and physical activity

Three studies provide information on the changes of the leisure and physical activity interventions on the *QoL* of informal caregivers. One study describes positive findings [65], while the other two report no significant improvement in *QoL* [63, 71].

##### Counselling

In six studies, counselling approaches are applied to improve the *QoL* of informal caregivers. Five support interventions result in non-significant results [75–79]. Only one study reports a significant increase in the intervention group versus control group [72].

##### Cognitive behavioural approaches

There is one study in the sample, focusing on the effects of a cognitive behavioural support intervention on the *QoL* of caregivers, without being able to report positive results [83].

##### Befriending and peer-support

Both studies investigate the intervention effects on *QoL* [63, 88]. Neither of them shows improvements for the informal caregivers in the intervention groups.

The overall results for the outcomes burden, depression and QoL are summarized in Table 4.

**Table 4:** Burden, depression, QoL and No. of significant effects

Category	Outcomes and No. of significant effects (total; %)		
	Burden	Depression	Quality of life
Psychoeducation	5/13 (38.5%)	4/11 (36.4%)	3/11 (27.3%)
Leisure & physical	3/4 (75%)	0/3 (−)	1/3 (33.3%)
Counselling	2/5 (40%)	1/4 (25%)	1/6 (16.7%)
Cognitive behavioural	2/4 (50%)	4/6 (66.7%)	0/1 (−)
Befriending & peer support	–	–	0/2 (−)
**Total**	**12/26 (46.2%)**	**9/24 (37.5%)**	**5/23 (21.7%)**

#### Other outcomes

In addition to the three major outcome concepts listed separately, the following target parameters were identified as mental health outcomes: *Stress* is analysed ten times, *well-being* eight times. *Anxiety* is studied nine times. A small number of studies investigate the concepts of *mood* (2) and *grief* (1). Altogether, significant effects are observed in eight out of these 30 cases (26.7%): Three times for *well-being*, also three times for *anxiety* and one time for *stress* and *grief* respectively.

##### Psychoeducation

In total there are nine studies reporting on other outcomes. Five of these studies show significant improvements for the intervention groups compared to the control groups [43, 47, 48, 53–55]. Interestingly, all three studies with the outcome *anxiety* describe significant effects [43, 48, 53, 54]. Two studies show positive effects for *well-being* [47, 55]. No significant results are detected for *stress*, *grief* and *mood*.

##### Leisure and physical activity

Other outcomes are examined in five leisure and physical activity studies [63, 64, 68, 70, 71]. None of the interventions included here reports significant beneficial effects.

##### Counselling

In four counselling interventions, other outcomes have been tested [74, 75, 77, 78]. As with the leisure and physical activity interventions, no positive effects are found.

##### Cognitive behavioural approaches

Four intervention programmes are addressing at least one of the other outcomes. Two of these interventions show no significant effects [85]. Another study reports positive effects on the parameter *stress*, but not for *well-being* [81, 82]. Positive impacts on the outcomes *well-being* and *grief* are described in one study [86, 87].

##### Befriending and peer-support

One study focuses on other outcomes (anxiety, stress), but does not report significant effects [63].

The results for the other outcomes, in numbers and percentage per content category, are summarized in Table 5.

**Table 5:** Other outcomes and No. of significant effects

Category	Other outcomes and No. of significant effects (total; %)				
	Well-being	Anxiety	Stress	Grief	Mood
Psychoeducation	2/4 (50%)	3/3 (100%)	0/1 (−)	–	0/1 (−)
Leisure & physical	0/2 (−)	0/2 (−)	0/4 (−)	–	0/1 (−)
Counselling	–	0/1 (−)	0/3 (−)	–	–
Cognitive behavioural	1/2 (50%)	0/2 (−)	1/1 (100%)	1/1 (100%)	–
Befriending & peer support	–	0/1 (−)	0/1 (−)	–	–
**Total**	**3/8 (37.5%)**	**3/9 (33.3%)**	**1/10 (10%)**	**1/1 (100%)**	**0/2 (20%)**

By changing the perspective from the outcomes to the interventions, it can be observed, that in twenty-five of the forty-six interventions analysed, positive effects on at least one of the outcomes examined are measured. This represents a share of 54.3%. With respect to the five content categories, the highest success rate is found for the cognitive behavioural approaches (85.7%); in six of seven studies, at least one positive effect is reported. For the leisure and physical activity interventions, the rate is 55.6%. Counselling approaches show effects in half of the interventions studied (50%), whereas psychoeducational interventions are at about 45%. No positive effects are reported for the two befriending and peer-support interventions.

### Subgroup orientation of intervention programmes

Our analyses indicate that subgroup orientation is very slightly manifested in this sample. There are only six out of forty-six intervention programmes explicitly focusing on a specific subgroup of informal dementia caregivers, defined by one of the socio-demographic characteristics mentioned above [45, 60, 64, 72, 74, 88]. In all other interventions, the group of caregivers is addressed more generically as a whole, without further differentiation according to certain life and care conditions. Additional file 3 gives an overview of the six subgroup oriented interventions and the characteristics tailored. The level of effectiveness of the tailored interventions is relatively low. Out of the nine mental health outcomes studied, only one outcome (QoL) shows positive effects for the informal caregivers in the intervention group [72].

## Discussion

Due to the diverse and intensive care work in difficult structural circumstances, informal caregivers are disadvantaged in terms of their chances for healthy ageing [11, 91, 92]. A number of studies show that mental health aspects are particularly affected [29, 38]. As previous reviews demonstrate, support programmes for informal caregivers of persons living with dementia can have positive impacts on mental health outcomes [93]. Therefore, regular updates of the exiting research evidence is important. The overall aim of this review was to systematically collect and review the current scientific evidence on this issue. We focused on three main issues: First, content and procedures of the applied interventions. Second, the interventions’ effectiveness on outcomes of informal caregiver’s mental health. Third, since studies suggest that this might lead to more effective results, the subgroup orientation of intervention programs was analysed [34, 89].

### Methodical quality of studies

The methodological quality of the included studies was rated following the criteria established by Brodaty et al. [9], which are based upon Cochrane Collaboration Guidelines [41]. Although only studies of high quality were included, the sample shows that there is still potential to further improve the quality of future research, for instance with regard to the realization of larger sample sizes to carry out detailed subgroup analyses or the conduction of long-term follow-ups (see Additional file 2). Long-term follow-ups (at least 6 months) are useful to assess whether intervention impacts can be maintained over time [94]. However, the implementation of a long-term follow-up may involve some challenges, such as noncompliance, treatment switching, co-intervention or loss to follow-up and death [95].

### Content of interventions

A comparison of the different support programmes should only be conducted with thorough consideration. The interventions are rather heterogeneous, both in terms of the actual content and features of programmes as well as the way they are implemented (i.e. in terms of recipients, intensity, duration, medium, outcomes). As shown above, most interventions (39 out of 44) studied involve direct physical contact. Under the current circumstances of the Covid-19 pandemic, with physical distancing a social priority, this points to significant challenges informal caregivers of persons living with dementia face. Challenges characterized by tensions between the need to limit physical contacts to protect risk groups and the need for close support to ensure a stable care situation at home [96]. In particular, the physical distancing regulations may have negative effects on the ability to maintain social relationships and social interactions and thus – due to a decline of social health – have adverse overall health impacts [97, 98]. Intervention studies also operate in the tension between “risk protection and close support”. Many approaches, even successful ones, cannot be implemented due to pandemic-related regulations. This requires the development of innovative approaches and sensitive reflection on the feasibility of digital alternatives, particularly with regard to the heterogeneity of the group of informal caregivers in terms of their digital literacy [99].

Most interventions are complex and have multiple and diverse components complicating comparisons between intervention programmes and precise conclusions on their effects on mental health outcomes [100]. This also applies to the measuring instruments used. Although dominant instruments are apparent for some of the outcomes (i.e. ZBI for burden, CES-D for depression), overall a large number of different instruments is used, which also makes it more difficult to compare the effects of the programmes. In addition, the focus and way of reporting on intervention studies differs substantially regarding content, structure or process, because existing classification systems for intervention reporting are still too rarely applied [100, 101]. Mostly the intervention studies focus on improvements regarding informal caregiver’s depression, burden and QoL, which underlines their pivotal role in the discourse as already highlighted in other reviews [36–38].

### Effectiveness of interventions

As no meta-analyses were carried out, the analyses of the statistical significance of intervention results should be treated with caution. Furthermore, one should bear in mind the limited clinical and practical validity of statistically significant results, to which the discussion on the clinical relevance of health interventions refers [102]. Nevertheless, positive significant results are reported for one third of all mental health outcomes observed. It can therefore be confirmed that effective interventions to promote the mental health of informal caregivers of persons living with dementia exist [9, 103]. However, it must also be noted that many of the statistically significant results were only observed directly after the end of the intervention and that in the majority of studies with a long-term follow-up those results are no longer existent at the last measurement point. While positive effects were found in about half (51.3%) of the outcomes examined in interventions with a pretest-posttest design, the proportion decreases to about a quarter (27.8%) in studies with a follow-up measurement of at least 6 months after the end of the intervention.

Interventions – even if subsumed under the same content category – differ considerably in regard to the applied methods, the specific contents and the way support is delivered. Therefore, it is not possible to deduce universal mechanisms or factors that cause positive effects on the mental health of caregivers. In addition, explanations of underlying mechanisms of action are rarely described in detail.

With only a few exceptions, the success rate of the intervention programmes is relatively low, irrespective of the content applied. Most frequently positive effects of interventions are reported on the outcome of *subjective caregiver burden* (46.2%, Table 4), which could be an argument for a stronger focus on this parameter in interventions to support mental health for informal dementia caregivers. Among the studies that aim to reduce *subjective caregiver burden*, leisure and physical activity approaches were the most effective [67–69] (Table 4). Although this conclusion is based on only four studies, the present analysis hereby confirms earlier results concerning the positive effects physical activity interventions show in reducing dementia caregiver burden [104]. There is limited information by the authors about the exact mechanisms of action of leisure and physical activity interventions. However, three mechanisms can be assumed to lead to positive effects on perceived burden. First, a direct effect through an improvement of caregivers’ physical health. Second, it is concluded that participating in the interventions is enjoyable and that this has a positive effect on perceived burden. A third mechanism describes leisure and physical activity interventions as being a temporary relief, helping caregivers to think about something different than their ubiquitous care work.

Psychoeducational interventions are also fairly successful in reducing the subjective burden of dementia caregivers, showing positive effects in 5 out of 13 cases [30, 42, 44, 47, 55]. Other studies also underline that psychoeducational approaches to mental health improvement are quite promising [10, 38]. Regarding mechanisms of action, authors conclude that psychoeducational programmes might positively influence the caregivers to adopt coping strategies that are problem-focused and foster social support seeking, both aspects being beneficial in terms of reducing the caregiver burden. Another suggestion is that psychoeducational interventions, often multi-modal, may target different drivers of caregiver burden and therefore may have more positive effects than other types of intervention. Consistent with the findings of Gallagher-Thompson [105], who reports a strong evidence-base for cognitive behavioural therapy interventions in promoting caregivers mental health, we conclude that cognitive behavioural intervention approaches are the most effective programmes to tackle depressive symptoms in informal caregivers of persons living with dementia [80–83, 85] (Table 4). Authors particularly highlight the following potential mechanisms of action: a) caregivers learn to apply and benefit from cognitive restructuring or reappraisal techniques and focus on positive gains and alternative thoughts; caregiver learn to focus on practices of self-care, relaxation, pleasant activities and communication of own needs and c) caregivers gain competence through knowledge teaching.

Only every fifth study points to positive effects on the outcome quality of life [30, 50, 51, 53, 54, 65, 72]. This could be related to broad multidimensional and stable nature of the QoL-concept, where positive effects are hard to detect. In addition, the high level of success of psychoeducational interventions for the outcome anxiety should be emphasized. Positive effects are evident in all three programs investigated [43, 48, 53, 54] (Table 5).

The reasons authors provide for interventions showing non-significant results on the relevant mental health parameters can also be important for the design of future research. Identified reasons are either related to the study’s methodology or the content and implementation of the intervention. In terms of methodology, for instance, some authors mention sample sizes too small to detect effects [49, 56, 66], or that it might have been more beneficial to include different outcomes or outcomes more sensitive to detect changes [56, 71]. With regard to content and implementation of the interventions, following aspects are stressed: inadequate target group orientation [52, 73, 78], short duration and/or low dosage of the intervention [56, 78], a failure to sufficiently address challenges due to a lack of complexity [52, 78], and lack of compliance by caregivers [73, 78].

### Subgroup orientation of interventions

A further intention of this study is to investigate whether intervention studies so far are sufficiently tailored to different informal caregiver target groups. As the results indicate, studies focusing on certain dementia caregiver subgroups report hardly any positive effects. Based on the analyses carried out in this review, we suggest that subgroup orientation is not adequately implemented in intervention studies focusing on mental health of informal caregivers of persons living with dementia. Studies show, that different life situations and care relationships require differentiated priorities in terms of support [28, 33, 34].

One of the reasons for the limited impact the subgroup-oriented interventions could be, that although the rationale for subgroup orientation is stressed by authors, the practical adaptation of interventions to meet specific subgroup needs seems inadequately implemented. For instance, interventions for adult-child caregivers should reflect multiple life-course responsibilities like balancing care and work by integrating the occupational setting both thematically and spatially into the intervention design. Another example is the adaptation of the medium used to deliver the intervention. Even today, older generations generally use digital media significantly less often than younger generations. This should also be reflected in the practical implementation of interventions, despite the current trend to put a strong focus on digital interventions. Otherwise, there is a risk to exclude certain subgroups from participating in intervention programmes [106].

### Strength and limitations

Some strengths of this review should be highlighted. The study is based on a broad systematic search strategy and we used four major international databases. In comparison to other studies, which focus only on one or a few outcomes, we included a broad range of psychological outcomes that have a key function in research about dementia caregiver mental health. In addition, this review assessed the subgroup orientation of the interventions included, something that has not yet been sufficiently taken into account. Nonetheless, this study has limitations. First, no review protocol was registered. The search for relevant articles was limited to electronic databases and no additional searches were conducted. This approach is transparent, traceable and facilitates the replicability of the search strategy. We focused the study selection on RCTs with good methodical quality to ensure that only most rigorous studies for assessing the efficacy of an intervention were included. We used criteria and rating established by Brodaty and colleagues [9] instead of the more rigorous risk of bias assessment developed by the Cochrane Collaboration Group [107]. We focused on literature published from 2009 to 2018 in English and German language. We have not pooled the data for a meta-analysis due to heterogeneity of studies. Thus, presentation of the results is descriptive with a narrative synthesis.

### Implications

A general conclusion about which types of interventions work best is not possible. In line with the conclusion Van’t Leven et al. draw, this review points out the necessity to carry out a detailed analysis of the intended target population prior to the design of an intervention [108]. This might lead to better fitting support programmes adapted to certain subgroups of informal caregivers of persons living with dementia. This concerns the structure and content as well as procedure of intervention programmes in order to improve the compatibility and thus increase the likelihood that interventions are beneficial for informal caregivers. This review therefore confirms the need for an increased scientific debate on the issue of the subgroup orientation of intervention programmes. It seems that it could be of considerable benefit for research and practice to target intervention studies and programmes more specifically towards the needs of different informal caregiver risk groups [34, 89]. In our opinion, this is the crucial finding of our study: A consistent orientation of intervention programmes towards certain subgroups might help to reduce the weaknesses of intervention studies so far. This requires, among other things, high-quality RCTs with larger samples and long-term follow up in order to be able to carry out differentiated and clinically relevant subgroup analyses.

Besides a sharpened subgroup orientation of interventions this study suggests, that it is also important to think carefully about the outcomes targeted. For instance, based on our results, we can conclude with caution that, leisure and physical activity interventions show good results when it comes to reducing the subjective burden of informal caregivers. For depressive symptoms, though, cognitive behavioural programmes show predominantly positive results.

## Conclusion

This review shows that intervention programmes can be beneficial for promoting the mental health of informal caregivers of persons living with dementia. In sum, cognitive behavioural approaches show promising results, especially concerning the reduction of depressive symptoms. Besides this, leisure and physical activity intervention and, with some limitations, psychoeducational approaches, seem to contribute to reducing subjective caregiver burden. Overall, most positive effects, in terms of the success rate (proportion of studies with positive effects compared to the total number of studies on the outcome) are reported for the outcome *subjective caregiver burden*. Even though there is already a number of studies on this issue, these results on caregiver burden indicate that further research is needed, particularly addressing the context of mental health interventions for dementia caregiver even stronger. In a methodical perspective, both longer follow-up intervals and larger samples should be pursued. The studies in this review show a limited focus on certain dementia caregiver subgroups. We would like to underline the potential that might be found in a consistent targeting of interventions to specific subgroups. There is a need for further research in this field.

## Supplementary Information

**Additional file 1.** The full search enquiry in PsycInfo database. This file shows enquiry, limitations and results of the literature search conducted.

**Additional file 2.** Interventions and their characteristics included in the systematic review. This file shows authors information, content category, implementation details (dosage, medium, features) and recipients of the interventions. Furthermore, intervention and control group sizes, length of follow-up, rating of methodical quality as well as mental health outcomes and instruments used are listed.

**Additional file 3.** Subgroup oriented interventions and the characteristics tailored. This file shows author information, the subgroup of caregivers focused, the rationale for subgroups focus given by the authors, intervention adaptations and results reported.

## Data Availability

Data sharing is not applicable to this article as no datasets were generated or analysed during the current study.

## Abbreviations

ADL: Activities of daily living
BDI: Beck Depression Inventory
BEHAVE-AD: Behavioural Pathology in Alzheimer’s Disease scale
BIZA-D: Berlin Inventory for caregivers’ burden with dementia patients
BPSD: Behavioural and psychological symptoms of dementia
CBI: Caregiver Burden Inventory
CES-D: Center for Epidemiologic Studies Depression Scale
CG: Control Group
CGS: Caregiver Grief Scale, CGS
CRA: Caregiver Reaction Assessment
CSI: Caregiver Strain Index
EQ-5D: European Quality of Life 5 Dimensions
F2f: Face to face
FCBI: Family Caregiving Burden Inventory
GHQ: General Health Questionnaire
HADS: Hospital Anxiety and Depression Scale
HDRS: Hamilton Depression Rating Scale
IADL: Instrumental activities of daily living
IC: Informal caregiver
IG: Intervention Group
MADRS: Montgomery–Åsberg Depression Rating Scale
NPI-D: Caregiver Distress Scale of the Neuropsychiatric Inventory
NPI-Q: Neuropsychiatric Inventory Questionnaire
PANAS: Positive and Negative Affect Schedule
PCI: Perceived Change Index
POMS: Profile of Mood States
PRISMA: Preferred Reporting Items for Systematic Reviews and Meta-Analyses
PSS: Perceived Stress Scale
QoL-AD: Quality of Life in Alzheimer’s Disease
RTC: Randomized controlled trial
RPWS: Ryff’s Psychological Well-Being Scale
RSS: Relative’s Stress Scale
SD: Standard deviation
SF 36: Short Form (36) Health Survey
SF-12 UK: Short Form (12) Health Survey United Kingdom Version
SPICC: Self-Perceived Pressure from Family Care
SRQ 20: Self Reporting Questionnaire 20-Item
VAS: Visual Analogue Scale
WHOQOL: World Health Organization Quality of Life
WHOQOL-BREF: World Health Organization Quality of Life Brief Version
ZBI: Zarit Burden Interview
