# Supplementary material for: Psychosocial interventions to support the mental health of informal caregivers of persons living with dementia – a systematic literature review
Source: BMC Geriatr. 2021 Feb 1;21:94. doi: 10.1186/s12877-021-02020-4 (PMC7849618; doi:10.1186/s12877-021-02020-4)
Supplement: Supplementary file 1 — Additional file 1. The full search enquiry in PsycInfo database. This file shows enquiry, limitations and results of the literature search conducted. [file 12877_2021_2020_MOESM1_ESM.docx]

Additional file 1: Full search enquiry in PsycInfo database

| **#** | **Enquiry** | **Limitations** | **Results** |
| --- | --- | --- | --- |
| 1 | dementia.sh. | limit to (human and abstracts and "0100 journal"  and (english or german) and yr="2009 - 2018") | 12496 |
| 2 | alzheimer*.sh. | limit to (human and abstracts and "0100 journal"  and (english or german) and yr="2009 - 2018") | 16360 |
| 3 | dementia.ti. | limit to (human and abstracts and "0100 journal"  and (english or german) and yr="2009 - 2018") | 10383 |
| 4 | dementia.ab. | limit to (human and abstracts and "0100 journal"  and (english or german) and yr="2009 - 2018") | 21972 |
| 5 | alzheimer*.ti | limit to (human and abstracts and "0100 journal"  and (english or german) and yr="2009 - 2018") | 11470 |
| 6 | alzheimer*.ab | limit to (human and abstracts and "0100 journal"  and (english or german) and yr="2009 - 2018") | 20303 |
| 7 | 1 or 2 or 3 or 4 or 5 or 6 |  | 35448 |
| 8 | caregiver*.sh | limit to (human and abstracts and "0100 journal"  and (english or german) and yr="2009 - 2018") | 11374 |
| 9 | caregiv*.ti | limit to (human and abstracts and "0100 journal"  and (english or german) and yr="2009 - 2018") | 5902 |
| 10 | caregiv*.ab | limit to (human and abstracts and "0100 journal"  and (english or german) and yr="2009 - 2018") | 18150 |
| 11 | care-giv*.ti | limit to (human and abstracts and "0100 journal"  and (english or german) and yr="2009 - 2018") | 104 |
| 12 | care-giv*.ab | limit to (human and abstracts and "0100 journal"  and (english or german) and yr="2009 - 2018") | 832 |
| 13 | carer*.ti | limit to (human and abstracts and "0100 journal"  and (english or german) and yr="2009 - 2018") | 1154 |
| 14 | carer*.ab | limit to (human and abstracts and "0100 journal"  and (english or german) and yr="2009 - 2018") | 4264 |
| 15 | "home care".ti | limit to (human and abstracts and "0100 journal"  and (english or german) and yr="2009 - 2018") | 720 |
| 16 | "home care".ab | limit to (human and abstracts and "0100 journal"  and (english or german) and yr="2009 - 2018") | 1878 |
| 17 | "home-based care".ti | limit to (human and abstracts and "0100 journal"  and (english or german) and yr="2009 - 2018") | 38 |
| 18 | "home-based care".ab | limit to (human and abstracts and "0100 journal"  and (english or german) and yr="2009 - 2018") | 181 |
| 19 | community-dwelling.ti | limit to (human and abstracts and "0100 journal"  and (english or german) and yr="2009 - 2018") | 1340 |
| 20 | community-dwelling.ab | limit to (human and abstracts and "0100 journal"  and (english or german) and yr="2009 - 2018") | 4430 |
| 21 | "domestic care".ti | limit to (human and abstracts and "0100 journal"  and (english or german) and yr="2009 - 2018") | 5 |
| 22 | "domestic care".ab | limit to (human and abstracts and "0100 journal"  and (english or german) and yr="2009 - 2018") | 7 |
| 23 | relatives.ti | limit to (human and abstracts and "0100 journal"  and (english or german) and yr="2009 - 2018") | 878 |
| 24 | couple*.ti | limit to (human and abstracts and "0100 journal"  and (english or german) and yr="2009 - 2018") | 3574 |
| 25 | spouse*.ti | limit to (human and abstracts and "0100 journal"  and (english or german) and yr="2009 - 2018") | 649 |
| 26 | 8 or 9 or 10 or 11 or 12 or 13 or 14 or 15 or 16 or 17 or 18 or 19 or 20 or 21 or 22 or 23 or 24 or 25 |  | 34561 |
| 27 | 7 and 26 |  | 4924 |
