# Supplementary material for: Psychosocial interventions to support the mental health of informal caregivers of persons living with dementia – a systematic literature review
Source: BMC Geriatr. 2021 Feb 1;21:94. doi: 10.1186/s12877-021-02020-4 (PMC7849618; doi:10.1186/s12877-021-02020-4)
Supplement: Supplementary file 2 — Additional file 2. Interventions and their characteristics included in the systematic review. This file shows authors information, content category, implementation details (dosage, medium, features) and recipients of the interventions. Furthermore, intervention and control group sizes, length of follow-up, rating of methodical quality as well as mental health outcomes and instruments used are listed. [file 12877_2021_2020_MOESM2_ESM.docx]

Additional file 2 Characteristics of interventions

| **Authors** | **Content**  **category** | **Implementation**  **(dosage, medium, features)** | **Recipient** | **Group**  **size** | **Follow-**  **up** | **Quality**  **Score** | **Mental health**  **outcomes** |
| --- | --- | --- | --- | --- | --- | --- | --- |
| **Psychoeducation (n=20)** | |  |  |  |  |  |  |
| Berwig et al. ^44^ | Psychoeducation | 12 sessions (f2f, 1h; phone, 30 min.) over 22 weeks; *features:* REACH II adaption, 5 modules, individual coaching: information, role-playing, problem-solving, coping with challenging behaviours, stress management techniques, social support, health behaviours | Informal caregiver (IC) | IG***: 47  CG: 45 | 3 month | 9 | Burden (Zarit Burden Interview, ZBI)  QoL (Short Form 36) |
| Blom et al. ^45^ | Psychoeducation | 9 sessions (web-based); *features:* coping with behavioural problems, relaxation, arranging help from others, cognitive reframing, communication; text material and videos, exercises and homework, evaluation at start and end of each session. | IC | IG: 149  CG: 96 | Post-test | 8 | Depression (CES-D)  Anxiety (Hospital Anxiety and Depression Scale, HADS)^**^  Burden (Self-Perceived Pressure from Informal Care, SPPIC) |
| Chen et al. ^46^ | Psychoeducation | 6 sessions (f2f) over 3 months; *features:* problem-solving skills, managing own emotions increasing knowledge of dementia and caregiver-support strategies | IC | IG: 24  CG: 22 | Post-test | 8 | Burden (ZBI Chinese Version) |
| Czaja et al. ^47^ | Psychoeducation | 11 sessions (videophone, f2f) over 5 months; *features:* videophone intervention, provision of education, support, and skill-building | IC | IG: 38  CGa: 36  CGb: 36 | Post-test | 8 | Depression (CES-D) |
| Gitlin et al. ^78^ | Psychoeducation | Up to 12 sessions (f2f, phone) over 4 months; *features*: caregiver education (capabilities, medications, pain), addressing concerns, problem-solving, communication, engaging in activities, simplifying tasks | Dyads | IG: 102  CG: 107 | 5 months | 10 | QoL (Quality of Life in Alzheimer's Disease, QoL-AD)  Well-being (Perceived Change Index, PCI) |
| Gitlin et al. ^48^ | Psychoeducation | Up to 11 sessions (f2f, phone) over 16 weeks; *features:* identifying potential triggers of patient behaviours (communication, environment, medical conditions), training in strategies to modify triggers and reduce upset | IC | IG: 117  CG: 122 | 3 months | 9 | Burden (ZBI)  Well-being (Perceived Change Index)  Depression (CES-D) |
| Judge et al. ^79^ | Psychoeducation | 6 sessions (f2f) over 15 weeks; *features:* education about dementia and memory loss, effective communication, managing memory, staying active, recognizing emotions and behaviours | Dyads | IG: 68  CG: 60 | Post-test | 8 | Anxiety (Zung Anxiety Scale)  Depression (CES-D)  QoL (QoL-AD) |
| Kunik et al. ^80^ | Psychoeducation | 6-8 sessions (f2f) over 8 weeks; features: targeting pain and distress, including mood problems, lack of engagement in activities, difficulty in communication | Dyads | IG: 101  CG: 102 | 9 months | 10 | Depression (Geriatric Depression Scale, GDS)  Burden (ZBI) |
| Kuo et al. ^49, 50^ | Psychoeducation | 20 sessions (f2f, phone) over 75 weeks; *features:* provision of skills and strategies to lower environmental stresses, emphasizing partnership between service providers and caregivers, help to identify stresses causing behavioural problems, develop environmental support and case-management services | IC | IG: 63  CG: 66 | Post-test | 9 | Depression (Chinese Version CES-D)  QoL (SF-36) |
| Kurz et al. ^81^ | Psychoeducation | 13 sessions (f2f) over 15 months; *features:* information about Alzheimer’s disease and is structured along the different stages of dementia severity | Dyads | IG: 156  CG: 136 | Post-test | 9 | Depression (Montgomery–Åsberg Depression Rating Scale, MADRS)  QoL (Short SF 36) |
| Livingston et al. ^51, 52^ | Psychoeducation | 8 sessions (f2f) over 4 months; *features:* education about dementia, carers’ stress, emotional support, understanding and managing behaviours, changing unhelpful thoughts, promoting acceptance, relaxation; planning the future, increasing pleasant activities | IC | IG: 173  CG: 87 | 20 months | 10 | Anxiety (HADS)  QoL (QoL-AD) |
| Martin-Carrasco et al. ^53^ | Psychoeducation | 8 sessions (f2f) over 16 weeks; *features:* information about dementia, elements of cognitive-behavioural guidance, such as training in the control of activation, cognitive restructuring techniques, problem solving and increasing rewarding activities | IC | IG: 60  CG: 55 | 6 months | 9 | Well-being (GHQ)  Burden (ZBI)  QoL (SF 36) |
| Martin-Carrasco et al. ^54^ | Psychoeducation | 7 sessions (f2f) over 14 weeks; *features:* standardized information about the clinical course of dementia, training on cognitive and behavioural skills and relaxation techniques, communicative skills, and emotional control | IC | IG: 115  CG: 123 | 4 months | 9 | Burden (ZBI)  Well-being (General Health Questionnaire, GHQ)  QoL (SF-12) |
| Prick et al. ^82^ | Psychoeducation | 8 sessions (f2f) over 12 weeks; *features:* physical exercise component (flexibility, strengthening,  balance, endurance exercises) and support component (education, communication skills training, pleasant activities training) | Dyads | IG: 57  CG: 54 | 3 months | 9 | Depression (CES-D)  Burden (Self-Perceived Pressure from Family Care, SPICC) |
| Rotrou et al. ^55^ | Psychoeducation | 12 sessions (f2f) over 12 weeks; *features:* education, problem-solving techniques, emotion-centred coping strategies, management of patient's behaviour, communication skills, crisis management, resource information, practical advice | IC | IG: 81  CG: 86 | 3 months | 9 | Burden (ZBI)  Depression (Montgomery and Asberg Depression Rating Scale, MADRS) |
| Soylemez et al. ^56^ | Psychoeducation | 3 sessions (f2f) over 3 months; *features:* understanding behaviours, care planning, understanding symptoms and causes of challenging behaviours, teaching of methods to modify the environment to reduce behavioural symptoms | IC | IG: 35  CG: 35 | 3 months | 9 | Burden (Caregiver Burden Inventory, CBI)  Depression (BDI)  QoL (WHOQOL-BREF) |
| Steffen et al. ^57^ | Psychoeducation | 22 sessions (video/DVD, phone) over 10 months; *features:* behavioural activation, management of disruptive behaviours, relaxation, didactic and experimental materials, maintenance of skills | IC | IG: 33  CG: 41 | 6 month | 9 | Depression (BDI)  Mood (PANAS) |
| Tang et al. ^58^ | Psychoeducation | 6 sessions (f2f, phone) over 12 months; *features:* skill-building program actively involving the caregivers, aiming to enable them to recognize existing potentials, develop and execute own strategies, regain control of caregiving | IC | IG: 22  CG: 21 | 3 month | 9 | Burden (ZBI)  Stress (NPI-Q) |
| Tremont et al. ^59^ | Psychoeducation | 16 sessions (phone) over 6 months; *features:* dementia education, emotional support, directing to appropriate resources, encouraging to attend to their physical, emotional, and social needs, and teaching strategies to cope with ongoing problems | IC | IG: 133  CG: 117 | Post-test | 8 | Burden (ZBI)  Depression (CES-D)  QoL (EQ-5D) |
| Wang et al. ^60^ | Psychoeducation | 8 sessions (f2f) over 6 months; *features:* information about dementia and care, development of social network, sharing and adaptation of emotional impacts of caregiving, learning about self-care and motivation, improvement of relationships, establishing formal support, problem solving skills | IC | IG: 40  CG: 40 | Post-test | 9 | Burden (Family Caregiving Burden Inventory, FCBI)  QoL (WHO-QoL) |
| **Leisure and physical activity (n=9)** | |  |  |  |  |  |  |
| Charlesworth et al.* ^61^ | Leisure & physical activity (RYCT) | RYTC (IGc): Up to 19 sessions (f2f) over 10 months; *features*: group reminiscence therapy, discussions, small group activity, handling objects, acting or improvisation, singing, cooking | IC or dyads | IGa: 48  IGb: 97  IGc: 97  CG: 47 | Post-test, max. 2 months | 9 | QoL (UK Short Form-12 Health Survey, UK SF-12)  QoL (EQ 5 + VAS)  Anxiety (HADS)  Stress (Caregiver Distress Scale of the Neuropsychiatric Inventory, NPI-D) |
| Connell et al. ^62^ | Leisure and physical  activity | 14 sessions (phone) over 6 months; *features:* telephone-based exercise intervention, fostering physical activity, individualized goal setting, enhancing self-efficacy | IC | IG: 86  CG 71 | 6 month | 8 | Depression (CES-D)  Stress (Perceived Stress Scale, PSS) |
| Danucalov et al. ^63^ | Leisure and physical  activity | 24 sessions (f2f) over 8 weeks; *features:* practice of yoga in combination with compassion meditation | IC | IG: 25  CG: 21 | Post-test | 9 | QoL (WHO Quality of Life Questionnaire, WHO-QOL-BREF) |
| Gitlin et al. ^83^ | Leisure and physical  activity | 8 sessions (f2f) over 16 weeks; *features:* using activities, managing situational distress, understanding behavioural symptoms, learning to simplify activities for future and how to use strategies to care challenges | Dyads | IG: 76  CG: 84 | 4 months | 10 | Depression (CES-D)  Burden (ZBI-Short Form) |
| Hirano et al. ^84^ | Leisure and physical  activity | 72 sessions (f2f) over 24 weeks; *features:* performing individually chosen physical leisure activities at home together with care recipients | Dyads | IG: 17  CG: 14 | Post-test | 8 | Burden (ZBI) |
| Lowery et al. ^85^ | Leisure and physical  activity | 60 sessions (f2f) over 12 weeks; *features:* individually tailored regimen of walking designed to become progressively more intensive | Dyads | IG: 67  CG: 64 | Post-test | 9 | Burden (ZBI)  Stress (NPI-Q)  Well-being (General Health Questionnaire) |
| Mahdavi et al. ^64^ | Leisure and physical  activity | 5 sessions (f2f) over 5 weeks; *features:* prayers and encouraging to pray, discussions about divine issues, using holy  books for treatment, illustration and relaxation techniques, self-confessing spiritual beliefs and experiences | IC | IG: 33  CGa: 32  CGb: 35 | Post-test | 8 | Burden (Caregiver Strain Index, CSI) |
| Moore et al. ^65^ | Leisure and physical  activity | 6 sessions (f2f, phone) over 6 weeks; *features:* education on how to monitor time spent in leisure activities, identification of enjoyable leisure activities, prioritizing activities, scheduling/participating in leisure activities, tracking of moods | IC | IG: 49  CG: 51 | 12 months | 10 | Depression (CES-D)  Mood (Positive and Negative Affect Schedule, PANAS) |
| Woods et al. ^86^ | Leisure and physical  activity | 12 sessions (f2f) over 12 weeks; *features*: focusing different themes (childhood, schooldays, working life, marriage, holidays, journeys) work in large and small groups, and a range of activities (art, cooking, physical re-enactment of memories, singing and oral reminiscence) | Dyads | IG: 268  CG: 220 | 7 months | 9 | Well-being (GHQ-28)  Anxiety (HADS)  Stress (Relative's Stress Scale, RSS)  QoL (EQ-5D) |
| **Counselling (n=8)** | |  |  |  |  |  |  |
| Brijoux et al. ^66^ | Counselling | 5-25 sessions (f2f, phone, web-based over 16 weeks; *features:* counselling by specifically qualified family companions, care-related counselling, strengthen emotional well-being, support use of care services | IC | IG: 39  CG: 37 | Post-test | 8 | QoL (12-Item Short Form survey, SF-12)  Burden (Berlin  Inventory for caregivers’ burden with dementia patients, BIZA-D) |
| Fortinsky et al. ^67^ | Counselling | 12 session (f2f) over 12 months; *features:* care consultation, managing dementia symptoms, accessing community support services | IC | IG: 54  CG: 30 | Post-test | 8 | Depression (CES-D)  Burden (ZBI) |
| Gaugler et al. ^91^ | Counselling | 6 sessions (f2f) over 4 months + ongoing counselling; *features:* individual and family counselling, support group participation, and ad hoc counselling | Family or IC | IG: 54  CG: 53 | Post-test | 9 | Stress (4-item Overload Scale, Pearlin et al., 1990) |
| Gavrilova et al. ^68^ | Counselling | 5 sessions (f2f) over 5 weeks; features: basic education about  dementia and specific training on managing problem behaviours | IC | IG: 30  CG: 30 | 6 months | 10 | QoL (WHOQOL-BREF)  Stress (Self Reporting Questionnaire 20-Item, SRQ 20)  Burden (ZBI) |
| Geschke et al. ^87^ | Counselling | 1 session (f2f); *features:* care counselling, information about dementia, reflection on communication, relationship, legal situation, support services | Dyads | IG: 54  CG: 30 | 18 months | 8 | Depression (Becks Depression Inventory, BDI)  QoL (SF-36) |
| Guerra et al. ^69^ | Counselling | 5 sessions (f2f) over 5 weeks; *features:* basic education about dementia and specific training on managing problem behaviours | IC | IG: 29  CG: 29 | 6 months | 10 | Burden (ZBI)  Stress (SRQ 20)  QoL (WHOQOL-BREF)  Stress (NPI-Q) |
| Joling et al. ^90^ | Counselling | 6 sessions (f2f) over 12 months; *features:* psycho-education, problem solving techniques, mobilizing the existing family networks to improve support | Family | IG: 96  CG: 96 | Post-test | 10 | Depression (CES-D)  Anxiety (HADS-A)  Burden (Caregiver Reaction Assessment, CRA)  QoL (SF-12) |
| Phung et al. ^88^ | Counselling | 18-20 sessions (f2f, phone) over 8-12 months; *features:* use of log books, group teaching courses by dementia experts, information folders about dementia and legal issues and resources for social support | Dyads | IG: 163  CG: 167 | 24 months | 11 | Depression (GDS)  QoL (EQ-VAS) |
| **Cognitive behavioural (n=6)** | |  |  |  |  |  |  |
| Au et al. ^70^ | Cognitive behavioural approaches | 8 biweekly sessions (phone, 15-20 min.) over 16 weeks; *features:* individual training for symptoms, behavioural changes, stress, pleasant event scheduling, communication | IC | IG: 51  CG: 45 | Post-test | 8 | Depression (Center for Epidemiologic Studies Depression Scale, CES-D) |
| Cheng et al.* ^71, 72^ | Cognitive behavioural approach | 4 sessions (f2f) over 2 months; *features:* cognitive reappraisal, controlling upsetting thoughts, enhancing self-efficacy | IC | IG: 34  CGa: 33  CGb: 36 | Post-test | 9 | Depression (Hamilton Depression Rating Scale, HDRS)  Burden (ZBI)  Stress (4-item Overload Scale, Pearlin et al., 1990)  Well-being (Ryff’s Psychological Well-Being Scale, RPWS) |
| Kamkhagi et al. ^73^ | Cognitive behavioural approaches | 14 sessions (f2f) over 14 weeks; *features:* psychodynamic group therapy, reinforcing resilience abilities  (getting in contact with feelings, thinking about relationship, maintain affectionate caring relationship, adaption to new situation | IC | IG: 20  CG: 17 | Post-test | 8 | Depression (BDI)  QoL (WHO-QoL)  Burden (ZBI) |
| Kwok et al. ^74^ | Cognitive behavioural approach | 9 sessions (phone) over 8 weeks; *features:* identification of BPSD, exploration, response and discussion of maladaptive thoughts, modification of maladaptive thoughts, exploration of effective and practical coping strategies | IC | IG: 20  CG: 22 | Post-test | 8 | Burden (ZBI) |
| Losada et al.* ^75^ | Cognitive behavioural approach | 8 sessions (f2f) over 8 weeks; *CBT (IGa) features:* cognitive restructuring, assertive skills, relaxation, increasing pleasant activities | IC | IGa: 42  IGb: 45  CG: 48 | 6 months | 9 | Depression (CES-D)  Anxiety (Tension-Anxiety subscale; Profile of  Mood States, POMS) |
| Losada et al.* ^75^ | Cognitive behavioural approach | 8 sessions (f2f) over 8 weeks; *ACT (IGb) features:* acceptance of aversive internal events and circumstances, choosing meaningful courses of action | IC | IGa: 42  IGb: 45  CG: 48 | 6 months | 9 | Depression (CES-D)  Anxiety (Tension-Anxiety subscale; Profile of  Mood States, POMS) |
| Meichsner et al. ^76^, Wilz et al. ^77^ | Cognitive behavioural approach | 12 sessions (phone) over 6 months; *features:* identification and expression of painful  thoughts and emotions, managing painful emotions, acceptance of thoughts and emotions, redefinition of the relationship, (re)activation of resources, adaptation to bereavement | IC | IG: 139  CG: 134 | 6 months | 9 | Grief (Caregiver Grief Scale, CGS)  Depression (CES-D)  Well-being (Visual Analog Scale, Wilz & Soellner 2016)  Burden (one item BEHAVE-AD) |
| **Befriending & Peer-support (n=2)** | |  |  |  |  |  |  |
| Charlesworth et al.* ^61^ | Befriending & Peer support (CSP) | CSP (IGa): 22 sessions (f2f + phone) over 8 months; *features:* one-to-one peer support from experienced caregivers, peer supporters listen, encourage and give moral support | IC or dyads | IGa: 48  IGb: 97  IGc: 97  CG: 47 | Post-test, max. 2 months | 9 | QoL (UK Short Form-12 Health Survey, UK SF-12)  QoL (EQ 5 + VAS)  Anxiety (HADS)  Stress (Caregiver Distress Scale of the Neuropsychiatric Inventory, NPI-D) |
| Laakkonen et al. ^89^ | Befriending & Peer-support | 8 sessions (f2f) over 8 weeks; *features*: shared  information and support, doing and discussing together, sharing experiences, receiving and  giving peer support, overcoming own limits, feeling togetherness | Dyads | IG: 67  CG: 69 | 9 months | 10 | QoL (Short SF 36) |
| *: In Charlesworth et al. 2016 and Losada et al. 2015, two separate intervention programs each are described and tested against a control group. Therefore, the studies both appear twice in this table.  **: The HADS scale is classified as a measuring tool for anxiety, according Cooper et al. ^15^.  ***IG=Intervention Group; CG=Control Group | | | | | | | |
