# Supplementary material for: Psychosocial interventions to support the mental health of informal caregivers of persons living with dementia – a systematic literature review
Source: BMC Geriatr. 2021 Feb 1;21:94. doi: 10.1186/s12877-021-02020-4 (PMC7849618; doi:10.1186/s12877-021-02020-4)
Supplement: Supplementary file 3 — Additional file 3. Subgroup oriented interventions and the characteristics tailored. This file shows author information, the subgroup of caregivers focused, the rationale for subgroups focus given by the authors, intervention adaptations and results reported. [file 12877_2021_2020_MOESM3_ESM.docx]

Additional file 3 Intervention programmes with subgroup orientation

| **Study** | **Subgroup** | **Rationale for subgroup focus**  **given by authors** | **Intervention**  **adaption** | **Results reported** |
| --- | --- | --- | --- | --- |
| Czaja et al. ^47^ | Ethnic  minorities | Barriers (transportation, insufficient support, lack of knowledge about services) limit caregivers from participating in support programs and accessing resources | Digital technology used, all materials translated (Spanish) | No effect on *depression* |
| Steffen et al. ^57^ | Women, cohabitating | Gender differences in caregiving response and preferences, unique stressors when living together, challenges to participate in distance services (transportation, time constraints) | Method of contact adapted via video & telephone coaching | No effects on *depression* and *mood* |
| Connell et al. ^62^ | Female  spouses | Spouses vulnerable to stress-related health consequences; female informal caregivers report higher levels of distress; women are less likely to exercise than men. Thus, older female spouse informal caregivers are at “triple risk” | Content of material (Video) used adapted | No effects on *stress* and *depression* |
| Brijoux et al. ^66^ | Living in rural areas | Situation for family informal caregivers is exacerbated in rural areas, where they are even more isolated and have fewer possibilities to receive professional support, making support from families even more important | Definition of target group adapted (support expanded to entire informal caregivers family) | Significant effect on *QoL*; no effect on *burden* |
| Gaugler et al. ^91^ | Adult-children  (A-C) | Adult-child informal caregivers suffer more on certain dimensions of stress than spouses due to multiple life-course responsibilities and change in role relationship, interventions may have different effects on spouses and adult-child caregivers | Counseling protocol adapted; A-C-specific support group offered | No effect on *stress* |
| Lakkonnen et al. ^89^ | Spouses | Marital relationship (…) important resource to overcome challenges of dementia | No further information given | No effect on *QoL* |
